# Factors associated with access to health services among people with long COVID in the Brazilian Amazon

**DOI:** 10.3389/fpubh.2024.1503907

**Published:** 2024-12-18

**Authors:** Amanda Loyse da Costa Miranda, Vanessa Ladyanne da Silva Costa, Ana Rosa Tavares da Paixão, Melissa Barbosa Martins, Sandra Helena Isse Polaro, Carlos Leonardo Figueiredo Cunha, Eliã Pinheiro Botelho, Andrey Oeiras Pedroso, Ana Cristina de Oliveira e Silva, Renata Karina Reis, Glenda Roberta Oliveira Naiff Ferreira

**Affiliations:** ^1^Graduate Program in Nursing at the Federal University of Pará (PPGENF/UFPA), Universidade Federal do Pará, Belém, Pará, Brazil; ^2^School of Nursing of Ribeirão Preto, University of São Paulo (EERP/USP), São Paulo, Brazil; ^3^Department of Clinical Nursing, Federal University of Paraíba (DENC-CCS-UFPB), João Pessoa, Paraíba, Brazil

**Keywords:** post-acute COVID-19 syndrome, access to health services, barriers to access to health and wellness, primary health care, COVID-19, Long COVID

## Abstract

**Background:**

Access to healthcare services for the population with long COVID is a challenge, as healthcare systems have been tasked with responding effectively to the extensive clinical heterogeneity of this disease.

**Objective:**

To analyze the factors associated with access to health services among people with long COVID in the Brazilian Amazon.

**Methods:**

This is a cross-sectional study using a quantitative method, conducted through an online survey between May 2023 and January 2024. The study included participants aged 18 years or older, residing in the northern region of Brazil, with a confirmed diagnosis of COVID-19 and who experienced long COVID. Participants completed an adapted version of the questionnaire on the Research Electronic Data Capture platform. Data were analyzed by multiple logistic regression.

**Results:**

A total of 364 people with long COVID participated in the study, of which only 167 (45.88%) had access to healthcare to treat the symptoms of this clinical condition. In the final multiple logistic regression model, only factors related to the need for services were associated with healthcare access. Participants with symptoms of dermatological alterations (AOR = 2.57; *p* = 0.01), a pre-COVID-19 diagnosis of chronic disease (AOR = 5.62; *p* = 0.04), those who treated their most severe COVID-19 infection with the assistance of a healthcare professional (AOR = 4.97; *p* = 0.01), and those who used antibiotics during their most severe COVID-19 infection (AOR = 3.24; *p* = 0.01) were more likely to access healthcare services for treating long COVID.

**Conclusion:**

Factors related to the need for services were the only ones associated in this population. It is important to know these aspects to identify the most affected populations and propose measures.

## Introduction

The COVID-19 pandemic, which emerged in December 2019, has infected millions of people worldwide, becoming one of the greatest global health challenges of this century (1, 2). Although the majority of individuals who contract COVID-19 fully recover, the World Health Organization (WHO) estimates that approximately 10 to 20% of patients, after overcoming the acute phase of the illness and clearing the virus, may continue to experience persistent symptoms that impact their health. This condition is referred to as long COVID (1, 2).

Studies indicate that the prevalence of long COVID can vary significantly, ranging from 3 to 93%, depending on the study’s definition and methodology (3, 4). In Brazil, it is estimated that among those who have recovered from COVID-19, approximately 5.6 million individuals will require healthcare for long COVID, with 81% of them needing to seek medical services (5).

In this context, healthcare systems must be equipped to manage long COVID, a condition characterized by a prolonged clinical course. Access to healthcare services is crucial to ensuring the well-being of affected individuals (6). However, there is a notable lack of awareness among healthcare professionals regarding this condition, coupled with significant barriers to healthcare access, which has been described as “complex, difficult, and exhausting” (7).

As was the case with the COVID-19 pandemic, the management of long COVID received a slow response from the Brazilian government, with the first technical note being published only a year after studies showed that the effects of the disease could extend beyond the acute phase (8–12). These structural social and healthcare challenges elevate the risk of long COVID within the population, particularly among more vulnerable groups (10).

The insufficient number of rehabilitation units and the lack of training programs for professionals on the topic, especially those working in primary care, can result in higher costs for healthcare systems and further hinder access to care, considering that patients are consulted or referred based on their symptomatology without a holistic or comprehensive approach (13–16).

Considering the precarious state of the healthcare system across all levels of care, the population’s low social indicators, and the lack of robust literature, it is essential to prioritize the assessment of individuals accessing healthcare services and to identify the primary long COVID symptoms that necessitate medical attention. In this context, the persistent difficulty in accessing healthcare services related to long COVID can be analyzed through the lens of Andersen’s “Behavioral Model of Health Services Use.” This model aims to understand the factors that influence healthcare access and service utilization, which are categorized into three components: predisposing factors (personal characteristics), enabling resources (personal and community resources), and need for care (individual perception) (17).

Given this context, the objective of the present article is to analyze the sociodemographic and clinical aspects related to the use of health services among people with long COVID in the Brazilian Amazon (northern region).

## Materials and methods

### Study design and setting

Observational, cross-sectional, and analytical study with a quantitative method, conducted through an online survey between May 2023 and January 2024.

The northern region of Brazil presents one of the lowest Human Development Indexes (HDI) in the country, ranging from 0.645 to 0.708. States like Amapá and Roraima lead the region with the highest HDI scores, while Amazonas and Pará report lower values. Although the region is rich in natural resources, disparities in access to public services and geographic isolation contribute to its lower human development performance, which reflects inequalities in areas such as health, education, and income (18, 19).

**Figure 1:**
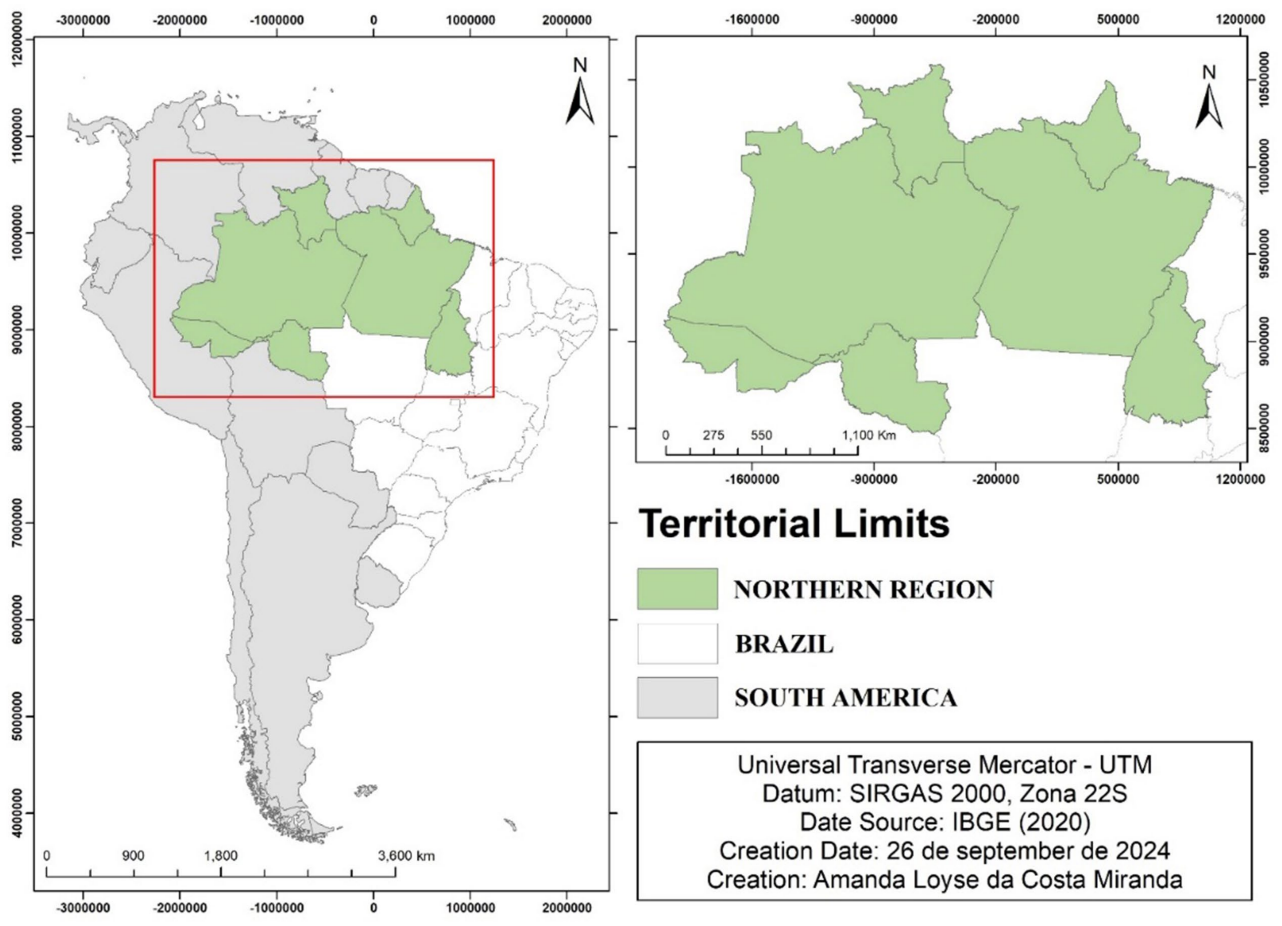
Northern region of Brazil. Source: Northern Region of Brazil © 2024 by Amanda Loyse da Costa Miranda is licensed under CC BY 4.0.

The population is predominantly composed of mixed-race individuals, and away from urban centers, there are groups of riverside communities, quilombola communities, and indigenous peoples who often live in geographic isolation due to the large territorial size of the states and insufficient transportation infrastructure. This affects socioeconomic development and hinders access to basic health services (Figure 1) (18, 19).

### Participants and selection criteria

The study included individuals aged 18 years or older, residing in the states of Acre, Amapá, Amazonas, Pará, Rondônia, Roraima, and Tocantins who self-reported having a laboratory-confirmed diagnosis of COVID-19 were included, and experienced the persistence of any physical and/or mental health symptoms for 4 weeks or more after COVID-19. The exclusion criteria were individuals without internet access or an electronic device to complete the questionnaire.

### Study size

The calculations were performed using the EpiInfo version 7.2.5.0 in the StatCalc module.

For the determination of the sample calculation, the following parameters were adopted: the number of recovered COVID-19 cases in the northern region, which totaled 2,827,897 accumulated cases (20–27); a long COVID case frequency of 34.9% (28); a margin of error of 5%; and a confidence interval of 95%, resulting in a minimum sample size of 349 participants.

After the sample calculation, a proportional stratification was carried out for each state in the northern region. The proportion of recovered COVID-19 cases was calculated for each state by dividing the number of recovered cases in that state by the total number of recovered cases in the northern region. Subsequently, the proportion of recovered COVID-19 cases for each state was multiplied by the initial sample calculation (349). The final sample consisted of: 104 (Pará), 20 (Acre), 22 (Amapá), 77 (Amazonas), 58 (Rondônia), 23 (Roraima), and 45 (Tocantins).

### Study instrument (questionnaire)

For data collection, we used an adapted version of the “Global COVID-19 Clinical Platform: Case Report Form (CRF) for Post COVID condition (Post COVID-19 CRF)” from the World Health Organization (WHO) (29). To adapt this instrument, we held five meetings with experts on the subject to define the information to be collected. Additionally, the research team modified the language used in the questions to make it more accessible to the general population. To enhance the fluency of the questionnaire, we minimized unnecessary reading effort, avoided redundancies, reduced the length of questions, and established a cohesive and coherent order for the questions. We also created a visually comfortable layout suitable for the various devices that participants might use (Supplementary material 1).

On average, participants took 15 min to complete the questionnaire. The questionnaire consisted of nine modules, but for this study, only four were utilized: demographic characterization, information related to SARS-CoV-2 infection, symptoms associated with long COVID, and use of health services. The questions in these domains were grouped according to the three main components of Andersen’s theory: predisposing factors, facilitating resources, and need for care/services.

In the predisposing factors, the following variables were used: Sex; Age; What is your highest level of education? Traditional Population; What is your current occupation? Skin color; Marital Status; Have you heard of the term Long COVID? Have you received any vaccines against COVID-19? How many doses? Did you receive a COVID-19 diagnosis before being vaccinated? How many times have you had a confirmed COVID-19 diagnosis?

In the facilitating resources, the following variables were used: Do you live in an Urban or Rural Area?; Individual income; Are you a beneficiary of Government Social Programs?

In the need for services component, the following variables were included: Before having COVID-19, did you have a diagnosis of any chronic disease?; Symptoms - Pain (General/Muscular/Joints); Symptoms Related to Mental Health (Fatigue/Suicidal Thoughts/Anxiety/Sleep Disturbances/Mood Changes/Depression/Stress/Suicide Attempt); Cardiovascular Symptoms (Heart Attack/Palpitations/Increased Heart Rate/Thrombosis/Arrhythmia); Dermatological Changes (Hair Loss/Dermatitis/Skin Changes); Loss of Taste; Loss of Smell; How would you rate your ability to care for yourself after the pandemic?; Regarding the episode you classified, how was your treatment/follow-up?; Thinking about your most severe COVID-19 infection, how would you classify it?; Did you receive Antibiotics?; Did you receive Antivirals?; Did you receive Ivermectin?; Did you receive Chloroquine?; Did you receive Home Remedies (herbal tea or bottled remedies)?; Respiratory Symptoms (Fatigue/Dyspnea/Shortness of Breath/Cough/Pulmonary Fibrosis/Pulmonary Embolism); Neurological Symptoms (Lack of Attention/Memory Loss-Forgetfulness/Headache/Dizziness); Symptoms of Non-Communicable Chronic Diseases (Hypertension/Diabetes); General Malaise Symptoms (Sweating/Tremors); Were you hospitalized for any of these symptoms?

In this study, we defined traditional populations of the Amazon as those living in the waterways, fields, and forests, including quilombola communities (Afro-descendants residing in specific territories), indigenous peoples, and riverside dwellers, in accordance with the concept established by the National Policy for Comprehensive Health of Rural and Forest Populations (30).

### Data collection

The study team was trained for data collection by experienced specialists in online survey research. Following this, data collectors (students from public and private universities in health-related undergraduate programs) were recruited through social media and college outreach. These individuals were properly trained for data collection through online meetings with the study team (master’s students and undergraduate research assistants). In total, seven universities in the state of Pará were visited, 51 online meetings were held with students from the northern region, and 257 data collectors were trained.

To reach the target population for the research, the Respondent Driven Sampling (RDS) methodology was employed, commonly used to access key populations that are hard to reach or when epidemiological data on a particular disease is not available (31). This methodology begins with a small group of participants, referred to as “seeds,” who contact individuals within their social circles to recruit new participants. These new participants, in turn, recruit others, forming chains or “waves” of recruitment, which allows for a more heterogeneous and representative sample of the population (31).

Thus, by adapting the method for virtual environments, each data collector contacted 10 individuals who had COVID-19 and sent the survey link via WhatsApp. After participants responded, each data collector requested the contacts of other individuals, thereby creating referral chains that extended the recruitment cycles or waves, increasing the number of participants (31, 32). Each data collector recorded in an Excel spreadsheet the number of participants they contacted and how many individuals were referred. This process continued until the researchers achieved the minimum sample size for the study.

Participants received a link via WhatsApp from the data collectors, containing a message written in accessible language with information about the nature and confidentiality of the research. By clicking the link, they were directed to the Research Electronic Data Capture (REDCap®) platform, where they accessed the Informed Consent Form (ICF) and the adapted questionnaire.

The questionnaires were hosted on the REDCap® platform, which is specifically designed for online data management. This platform provides features and tools that enhance organizational efficiency and ensure secure data storage. It is important to highlight that, given the nature of the online survey, all the information collected was self-reported.

### Variables

The outcome variable of the study is based on the question ‘Did you seek any healthcare service to treat these symptoms?’, with ‘yes’ or ‘no’ as response options. This variable is binary in nature, with the event of interest being the ‘yes’ responses. The independent variables are those present in the questionnaire.

### Statistical analysis

The data collected were extracted from the REDCap® platform and exported to Microsoft Excel®, where descriptive statistics (absolute and relative frequencies) were calculated. Responses defined as no information (NI) were excluded from the analyses, and no percentage was calculated.

The primary hypothesis of the study, ‘there is an association between clinical and demographic aspects and the use of healthcare services among people with long COVID,’ was tested using multiple logistic regression, where the independent variables were associated with the variable ‘Did you seek any healthcare service to investigate and/or treat these symptoms?’ Given the dichotomous nature of the dependent variable, univariate logistic regression model was initially used to assess the association between the independent and dependent variables.

Variables with a *p*-value <0.20 were selected. The variable ‘Mental Health-Related Symptoms’ had only positive responses, making its inclusion in the regression impossible.

The selected variables were analyzed through multiple logistic regression using the stepwise backward method, which involves using the base model with all previously inserted variables and then removing factors step by step to achieve the lowest Akaike Information Criterion (AIC).

Finally, variables with *p* > 0.05 were excluded from the final model. To assess the model’s quality, Odds Ratio (OR), Confidence Interval (CI), Akaike Information Criterion (AIC), and Variance Inflation Factor (VIF) were used. The analyses were performed using RStudio software version 2024.04.1 + 748, utilizing the following packages: base; readxl; dplyr; caret; MASS; car; rcompanion, and DescTools.

### Ethical approval and consent to participate

All requirements set forth by Resolution 466 of 2012 of the National Health Council of Brazil were followed, along with the principles established in the General Personal Data Protection Law No. 13,709 of 2018, particularly regarding personal data processing activities, as detailed in articles 6 and 7. The Declaration of Helsinki was also adhered to. The study received approval from the Research Ethics Committee of the Federal University of Paraíba, Lauro Wanderley University Hospital, under protocol number 5.826.893 and CAAE: 65929522.1.0000.5183.

All participants signed the Free and Informed Consent Form. The authors did not conduct experiments involving humans and/or use human tissue samples or human data. Additionally, there was no direct contact between the authors and the study participants, as the questionnaire was administered online, and the data were securely stored in the REDCap® platform, which ensures privacy and data security.

## Results

### Sociodemographic characteristics

This study included 364 people with long COVID, of whom only 167 (45.88%) sought healthcare services to treat the symptoms of this clinical condition. The average age of participants was 35.19 years (SD = 13.31), with 48% (173) in the age range of 18 to 32 years, 60% (217) being women, 63% (226) reporting they were not in a stable union/marriage, 70.8% (257) self-identifying as mixed race, Black, or Indigenous, and 89% (325) not being part of any traditional populations (quilombola/Indigenous/riverside) (Table 1).

**Table 1:** Association between predisposing characteristics and access to healthcare to treat Long COVID.

Predisposing factors	Access to healthcare to treat long COVID?		Total (364) *n* (%)	Regression		
	No (197) *n* (%)	Yes (167) *n* (%)		OR	95% CI	*p* value
Sex						
Female	101 (46.5)	116 (53.5)	217 (60)	2.16	(1.41; 3.34)	0.01
Male	96 (65.3)	51 (34.7)	147 (40)		Ref	
Age						
18–32	104 (60.1)	69 (39.9)	173 (48)	1.69	(1.11; 2.59)	0.01
33–47	64 (51.6)	60 (48.4)	124 (34)			
>47	29 (43.3)	38 (56.7)	67 (18)			
Education level						
High school or below	69 (57.5)	51 (42.5)	120 (33)		Ref	
Higher education	128 (52.5)	116 (47.5)	244 (67)	1.22	(0.79; 1.91)	0.36
Current occupation						
Unemployed/Student/Retiree	73 (54.9)	60 (45.1)	133 (37)		Ref	
Employee/Self-employed	119 (53.1)	105 (46.9)	224 (63)	1.07	(0.70; 1.65)	0.75
NI	5	2	7			
Skin color						
Yellow/White	59 (55.7)	47 (44.3)	106 (29)		Ref	
Indigenous/Brown/Black	137 (53.3)	120 (46.7)	257 (71)	1.1	(0.70; 1.74)	0.68
NI	1	0	1			
Marital status						
Married/Stable Union	67 (49.3)	69 (50.7)	136 (37)		Ref	
Single/Divorced/ Separate/ Widower	129 (57.1)	97 (42.9)	226 (63)	0.73	(0.47; 1.12)	0.15
NI	1	1	2			
Traditional population						
Yes	19 (48.7)	20 (51.3)	39 (11)	1.13	(0.58; 2.21)	0.71
No	177 (54.5)	148 (45.5)	325 (89)		Ref	
NI						
Number of confirmed COVID-19 diagnoses						
01	135 (57.2)	101 (42.8)	236 (65)		Ref	
02 or more	62 (48.4)	66 (51.6)	128 (35)	1.42	(0.92; 2.19)	0.11
COVID-19 diagnosis before being vaccinated?						
Yes	93 (47.7)	102 (52.3)	195 (56)	1.64	(1.07; 2.52)	0.02
No	91 (59.9)	61 (40.1)	152 (44)		Ref	
NI	13	4	17			
Have you received the COVID-19 vaccine?						
Yes	190 (53.7)	164 (46.3)	354 (98)		Ref	
No	4 (66.7)	2 (33.3)	6 (2)	0.58	(0.08; 3.0)	0.53
NI	3	1	4			
How many doses of vaccine?						
Up to 02 doses	28 (43.7)	36 (56.3)	64 (18)	1.63	(0.94; 2.82)	0.08
03 doses or more	162 (55.9)	128 (44.1)	290 (82)		Ref	
NI	7	3	10			
Have you heard of the term long COVID?						
Yes	23 (40.4)	34 (59.6)	57 (16)	1.97	(1.11;3.53)	0.02
No	173 (57.1)	130 (42.9)	303 (84)		Ref	
NI	1	3	4			

Northern Region, Brazil, 2024.

Source: Prepared by the author, 2024. *variable dichotomized as youth and adult. Ref, reference; OR, odds ratio; CI, confidence interval; SI, no information.

### Predisposing factors for accessing healthcare to treat long COVID

Table 1 presents the results of univariate logistic regression model showing the association between predisposing factors for accessing healthcare to treat long COVID in northern Brazil. The results show that female participants were twice as likely to access healthcare for long COVID (AOR = 2.16; *p* = 0.01). Participants already familiar with long COVID were nearly twice as likely to seek healthcare to treat its related symptoms (AOR = 1.97; *p* = 0.02).

Univariate logistic regression model showed that being aged 18 to 32 years (AOR = 1.69; *p* = 0.01) and being diagnosed with COVID-19 before vaccination (AOR = 1.64; *p* = 0.02) were significant factors. However, education level, marital status, current occupation, skin color, belonging to traditional populations, the number of COVID-19 diagnoses, receiving a vaccine, and the number of doses were not associated with accessing healthcare.

### Facilitating resources for accessing healthcare to treat long COVID

Regarding facilitating resources (Table 2), the results show that individuals with higher personal income (AOR = 1.53; *p* = 0.04) were more likely to access healthcare to treat long COVID. Being a beneficiary of government income distribution programs and residential areas did not influence healthcare access.

**Table 2:** Association between enabling resources and access to healthcare to treat Long COVID.

Facilitating resources	Access to healthcare to treat long COVID?		Total (364) *n* (%)	Regression		
	No (197) *n* (%)	Yes (167) *n* (%)		OR	95% CI	*p* value
Area of residence						
Rural area	16 (47.1)	18 (52.9)	34 (10)	1.12	(0.36; 2.23)	0.39
Urban and capital	177 (54.8)	146 (45.2)	323 (90)		Ref	
NI	4	3	7			
Individual income (in minimum wages)*						
1 MW or less	111 (59)	77 (41)	188 (53)		Ref	
2 or more MW	82 (48.5)	87 (51.5)	169 (47)	1.53	(1.01; 2.33)	0.04
NI	4	3	7			
Beneficiary of social programs?						
Yes	37 (56.9)	28 (43.1)	65 (18)		Ref	
No	155 (53.6)	134 (46.4)	289 (82)	1.14	(0.66; 1.98)	0.63
NI	5	5	10			

Northern Region, Brazil, 2024.

Source: Prepared by the author, 2024. *minimum wage in Brazil in September 2024 – R$ 1,412.00 – (US$ 262.67 – dollar exchange rate for the same period). Ref, reference; OR, odds ratio; CI, confidence interval; NI, no information; MW, minimum wage.

### Need for services to access healthcare for long COVID

In terms of service needs (Table 3), participants with a chronic disease diagnosis prior to COVID-19 were twice as likely to access healthcare for long COVID (AOR = 2.44; *p* = 0.01). Regarding medication therapy, participants who received antibiotics for treating a more severe COVID-19 infection were three times more likely to access healthcare (AOR = 3.77; *p* = 0.01). Additionally, participants who received antivirals (AOR = 2.57; *p* = 0.01), ivermectin (AOR = 2.19; *p* = 0.01), and chloroquine (AOR = 2.32; *p* = 0.01) were twice as likely to access healthcare.

**Table 3:** Association between the need for services and access to health care to treat Long COVID.

Need for Services	Access to healthcare to treat long COVID?		Total (364) *n* (%)	Regression		
	No (197) *n* (%)	Yes (167) *n* (%)		OR	95% CI	*p* value
Diagnosis of chronic disease before COVID-19						
Yes	21 (35.6)	38 (64.4)	59 (16)	2.44	(1.38; 4.42)	0.01
No	174 (57.4)	129 (42.6)	303 (84)		Ref	
NI	2	0	2			
Classification of the most severe COVID-19 infection						
Mild/Moderate	196 (54.9)	161 (45.1)	357 (98)		Ref	
Serious	1 (16.7)	5 (83.3)	6 (2)	6.0	(0.97; 117.25)	0.1
NI	0	1	1			
How was the monitoring/treatment of the most serious COVID-19 infection?						
I treated it with the supervision of a health professional	148 (50.7)	144 (49.3)	292 (83)	3.06	(1.64; 6.0)	0.01
I treated it myself. Without a health professional	44 (75.9)	14 (24.1)	58 (17)		Ref	
NI	5	9	14			
Did you receive antibiotics?						
Yes	104 (43.5)	135 (56.5)	239 (66)	3.77	(2.36; 6.14)	0.01
No	93 (74.4)	32 (25.6)	125 (34)		Ref	
Did you receive antivirals?						
Yes	69 (41.8)	96 (58.2)	165 (46)	2.57	(1.68; 3.97)	0.01
No	124 (64.9)	67 (35.1)	191 (54)		Ref	
NI	4	4	8			
Did you receive ivermectin?						
Yes	86 (45.3)	104 (54.7)	190 (53)	2.19	(1.43; 3.37)	0.01
No	107 (64.5)	59 (35.5)	166 (47)		Ref	
NI	4	4	8			
Did you receive chloroquine?						
Yes	23 (37.1)	39 (62.9)	62 (17)	2.32	(1.33; 4.14)	0.01
No	170 (57.8)	124 (42.2)	297 (83)		Ref	
NI	4	4	8			
Did you receive home remedies (herbal tea or bottle)?						
Yes	79 (49.4)	81 (50.6)	160 (44)	1.44	(0.95; 2.19)	0.08
No	118 (58.4)	84 (41.6)	202 (56)		Ref	
NI	0	2	2			
Symptoms-pain (General/Muscular/Joints)						
Yes	122 (48.6)	129 (51.4)	251 (77)	1.45	(0.87; 2.45)	0.16
No	44 (57.9)	32 (42.1)	76 (23)		Ref	
NI	31	6	37			
Symptoms related to mental health						
Yes	138 (49.1)	143 (50.9)	281 (100)	-	-	-
No	0 (0)	0 (0)	0			
NI	59	24	83			
Cardiovascular symptoms						
Yes	37 (36.6)	64 (63.4)	101 (48)	2.27	(1.31; 3.97)	0.01
No	63 (56.8)	48 (43.2)	111 (52)		Ref	
NI	97	55	106			
Dermatological changes						
Yes	58 (38.9)	91 (61.1)	149 (66)	2.46	(1.40; 4.35)	0.01
No	47 (61)	30 (39)	77 (34)		Ref	
NI	92	46	138			
Respiratory symptoms						
Yes	152 (50.7)	148 (49.3)	300 (97)	1.95	(0.5; 9.36)	0.35
No	6 (66.7)	3 (33.3)	9 (3)		Ref	
NI	39	16	55			
Neurological symptoms						
Yes	133 (50)	133 (50)	266 (90)	1.33	(0.61; 2.98)	0.47
No	16 (57.1)	12 (42.9)	28 (10)		Ref	
NI	48	22	70			
Symptoms of chronic non-communicable diseases						
Yes	27 (43.5)	35 (56.5)	62 (30)	1.28	(0.7; 2.34)	0.42
No	71 (49.7)	72 (50.3)	143 (70)		Ref	
NI	99	60	159			
Symptoms of general malaise						
Yes	73 (47.4)	81 (52.6)	154 (64)	1.5	(0.88; 2.55)	0.13
No	50 (57.5)	37 (42.5)	87 (36)		Ref	
NI	74	49	123			
Loss of smell						
Yes	110 (56.7)	84 (43.3)	194 (77)	0.5	(0.27; 0.91)	0.02
No	23 (39.7)	35 (60.3)	58 (23)		Ref	
NI	64	48	112			
Loss of taste						
Yes	100 (55.2)	81 (44.8)	181 (73)	0.68	(0.38; 1.18)	0.17
No	31 (45.6)	37 (54.4)	68 (27)		Ref	
NI	66	49	115			
Hospitalized for any of the symptoms of long COVID?						
Yes	3 (13.1)	20 (86.9)	23 (6.4)	8.67	(2.9; 37.3)	0.01
No	190 (56.5)	146 (43.5)	336 (93.6)		Ref	
NI	5	1	5			
How do you rate your ability to take care of yourself after the pandemic?						
Same/Better	27 (43.5)	35 (56.5)	62 (30)		Ref	
Worse	71 (49.7)	72 (50.3)	143 (70)	1.32	(0.79; 2.19)	0.28
NI	99	60	159			

Northern Region, Brazil, 2024.

Source: Prepared by the author, 2024. Ref, reference; OR, odds ratio; CI, confidence interval; NI, no information.

Participants who treated their more severe COVID-19 infection under the supervision of a healthcare professional were three times more likely to access healthcare for long COVID (AOR = 3.06; *p* = 0.01). Furthermore, participants hospitalized due to any long COVID symptoms had eight times the likelihood of accessing healthcare (AOR = 8.67; *p* = 0.01). A lower odds ratio in participants with loss of smell indicates that they were less likely to access healthcare (AOR = 0.5; *p* = 0.02).

### Components of healthcare access for treating long COVID: multiple logistic regression model

A multiple logistic regression (stepwise backward) was conducted to identify access components. All variables with a *p*-value <0.20 were selected and tested together in the model, including predisposing characteristics (‘sex,’ ‘how many vaccine doses?’ ‘How many times have you had a confirmed COVID-19 diagnosis?’), facilitating resources (‘income’), and service needs (‘chronic disease diagnosis before COVID-19,’ ‘classification of the most severe COVID-19 infection,’ ‘how was the follow-up/treatment of the most severe COVID-19 infection?’ ‘Received antibiotics?’ ‘Received antivirals?’ ‘Received ivermectin?’ ‘Received chloroquine?’ ‘Received home remedies [herbal teas or concoctions]?’ ‘Symptoms - pain [general/muscular/joint],’ ‘cardiovascular symptoms,’ ‘dermatological changes,’ ‘general malaise symptoms,’ ‘loss of smell,’ ‘loss of taste,’ ‘hospitalized due to any long COVID symptoms?’).

The final analysis model (Table 4) showed that participants with dermatological changes had twice the likelihood of accessing healthcare to treat long COVID (AOR = 2.57; *p* = 0.01); participants with a chronic disease diagnosis before COVID-19 were five times more likely to access healthcare for long COVID (AOR = 5.62; *p* = 0.04); participants who treated their more severe COVID-19 infection with healthcare professional supervision had almost five times the likelihood of accessing healthcare for long COVID (AOR = 4.97; *p* = 0.01) compared to those who treated it on their own; and participants who used antibiotics during their more severe COVID-19 infection were three times more likely to access healthcare for long COVID (AOR = 3.24; *p* = 0.01).

**Table 4:** Multiple logistic regression analysis of the associations between the components of access to healthcare to treat long COVID.

Variables	VIF	AOR	95% CI	*p*-value
Dermatological changes? (Ref. = no)				
Yes	1.03	2.57	(1.25; 5.39)	0.01
Diagnosis of chronic disease before COVID-19? (Ref. = No)				
Yes	1.01	5.62	(1.79; 22.58)	0.04
Regarding the episode you classified, how was your treatment/follow-up? (Ref. = I treated it alone, without a health professional)				
I treated it with the supervision of a health professional	1.04	4.97	(1.60; 18.47)	0.01
Did you receive antibiotics? (Ref. = No)				
Yes	1.01	3.24	(1.53; 7.06)	0.01

Northern Region, Brazil, 2024.

Source: Prepared by the author, 2024. AOR, Adjusted Odds Ratio; Ref, reference; CI, confidence interval; NI, no information.

## Discussion

In the context of limited specialized services for treating long COVID in the northern region of the country and low primary healthcare coverage, less than half of the participants (45.88%) sought healthcare services to address the symptoms of this clinical condition. The northern region of Brazil includes several municipalities with the worst sociodemographic indicators in the country. These characteristics of social and healthcare structure increase the risk of the population experiencing long COVID cases, particularly in more vulnerable populations (8–10).

In the final multiple logistic regression model, despite the inclusion of other variables related to predisposing factors and facilitating resources, only the factors related to the need for services were associated with healthcare access for treating long COVID. These factors included having a chronic disease diagnosed prior to COVID-19, receiving antibiotics, follow-up care for the most severe COVID-19 case, and dermatological conditions.

In the present study, participants with a chronic disease diagnosed prior to COVID-19 were more likely to access healthcare for treating long COVID (OR = 5.62; *p* = 0.04). Studies show that the presence of comorbidities significantly increases the risk, severity, and persistence of symptoms, as well as the prevalence of long COVID among those with multiple comorbidities (8, 14, 15, 33).

A study conducted in the United Kingdom involving over 486,000 adults observed that the main risk factors for long COVID are female sex, belonging to an ethnic minority, socioeconomic deprivation, and the presence of comorbidities (34). Another study conducted in southeastern Brazil showed that the prevalence of long COVID is higher among participants with chronic conditions and those who were hospitalized due to COVID-19, making them more likely to seek healthcare services (16). In Israel, hypertension emerged as a significant risk factor, particularly among younger patients (35).

Given that comorbidities are significant risk factors for poor clinical outcomes in COVID-19 (36), it is consistent that patients with a prior diagnosis of chronic diseases seek healthcare services to treat long COVID, highlighting the need for targeted healthcare strategies and strengthening comorbidity screening and control programs in primary healthcare (such as Hiperdia). Rehabilitation centers for treating long COVID across Latin America remain scarce (37).

The research shows that participants who had professional healthcare follow-up during their most severe COVID-19 infection were more likely to access healthcare for long COVID treatment (OR = 4.97; *p* = 0.01) compared to those who managed their condition alone. This aligns with studies indicating that individuals with more severe COVID-19 infections are at higher risk of developing long COVID. These same studies suggest that patients previously hospitalized for COVID-19 more frequently use healthcare services and report a higher number of symptoms than individuals with mild acute infection (15, 16, 38). This result underscores the need for continuous, multidisciplinary monitoring to manage the complexity of multiple symptoms and uncertain prognosis related to the disease, ensuring adequate access to healthcare.

Regarding medication therapy, participants who used antibiotics during their most severe COVID-19 infection were more likely to access healthcare for long COVID treatment. In contrast, a study conducted in the United States showed that patients who had early access to medications during the onset of the pandemic were more likely to discontinue treatments, reducing the flow of new patients into healthcare services (39).

Studies have shown that the use of antibiotics during the COVID-19 pandemic to treat patients was primarily empirical, often without proven efficacy, and that disordered use could worsen antibiotic resistance and increase the risk of adverse reactions (40). A systematic review indicates that antibiotics should not be prescribed during COVID-19 unless there is a clinical suspicion of coinfection (41).

Participants with symptoms related to dermatological conditions (such as hair loss, dermatitis, and skin changes) were more likely to access healthcare for treating long COVID (OR = 2.57; *p* = 0.01). Hair loss is a common symptom in patients with long COVID (15, 33, 34, 38, 42, 43).

A meta-analysis revealed that hair loss is present in almost 80% of patients with long COVID (44). Another study shows that about 20% of patients may continue to experience this symptom for more than 6 months after the initial COVID-19 infection (45), which may affect the patient’s self-image and quality of life in the long term.

Barriers to accessing healthcare services for long COVID treatment are multifaceted (14). The lack of protocols for addressing specific symptoms, such as dermatological factors, discourages patients from continuing treatment within the healthcare system (15, 34, 43). Research suggests that healthcare professionals often struggle to associate and recognize the heterogeneity of long COVID symptoms (14, 15, 38).

Patients report that symptoms described to professionals are often not recognized, leading to negative experiences with healthcare services (46). Furthermore, logistical challenges, such as long wait times for appointments, geographic accessibility, and limited access to specialized clinics, impact healthcare equity (37). Additionally, ethnic minorities experience distrust and fear of services due to social and structural restrictions that affect access to primary care services (8, 9, 46).

The results presented highlight the urgency of developing and implementing rehabilitation systems to support the adequate recovery of the affected population, given the delay in establishing guidelines for the care of long COVID patients (37, 43, 46). Given the particularities of the study population, delays in implementing care may further exacerbate regional inequalities, considering the complexity of access barriers across the region (47). Understanding that long COVID follow-up is important for improving patients’ quality of life, more scientific evidence is needed to understand its causes, barriers to healthcare access, mechanisms, and risks (48, 49).

### Strengths and limitations

The study’s limitations are related to the need for confirmed COVID-19 diagnosis through rapid tests or laboratory exams (limiting participant recruitment) and the inherent limitations of online surveys, such as the homophily and self-selection phenomenon. The first refers to participants with similar sociodemographic conditions, and the second to the lack of control over respondents, which influenced the average age, income, and education level of the study participants. Moreover, the use of electronic questionnaires may have limited the participation of older individuals and those with lower levels of education.

It is crucial to acknowledge that the cross-sectional observational design of this study does not facilitate the establishment of causal relationships between dependent and independent variables. Consequently, the characteristics of the population and the healthcare system examined in this study should be taken into account when generalizing the findings.

The web survey allowed us to understand access to health services among Amazon residents who had symptoms of long COVID, which would be difficult and costly if the questionnaire were applied in person. However, the limitation was having the self-report of the laboratory diagnosis of COVID-19.

## Conclusion

Ensuring access to health services is essential in the context of long COVID. Among the components of access, only factors related to the need for services were associated with accessing care. The study demonstrated that individuals with long COVID who experienced dermatological conditions, had a chronic disease diagnosis prior to COVID-19, received health follow-up, and used antibiotics during the COVID-19 infection were more likely to seek healthcare. Therefore, understanding the factors that influence the components of access is vital for establishing care guidelines and identifying vulnerable populations.

## Data Availability

The raw data supporting the conclusions of this article will be made available by the authors, without undue reservation.
