# Supplementary material for: Factors associated with access to health services among people with long COVID in the Brazilian Amazon
Source: Front Public Health. 2024 Dec 18;12:1503907. doi: 10.3389/fpubh.2024.1503907 (PMC11688473; doi:10.3389/fpubh.2024.1503907)
Supplement: Supplementary file 1 [file Data_Sheet_1.pdf]

INFORMED CONSENT

FREE AND INFORMED CONSENT FOR ONLINE DATA COLLECTION

You are being invited to participate in the research entitled "Prevalence and Quality of Life in Long COVID: Evidence for Educational Health Interventions," coordinated by Prof. Dr. Renata Karina Reis from the Graduate Program in Fundamental Nursing at the Ribeirão Preto School of Nursing, University of São Paulo, Prof. Dr. Glenda Roberta Oliveira Naiff Ferreira from the Federal University of Pará, and Prof. Dr. Ana Cristina de Oliveira e Silva from the Department of Clinical Nursing at the Federal University of Paraíba.
The aim of the research is to analyze the prevalence and the physical and mental consequences experienced over a prolonged period after COVID-19 infection in the general population to support educational health interventions that improve the quality of life for people affected by these repercussions.
Type of Participation: If you agree to participate, you will need internet access to complete the online questionnaire for this study. The research includes personal and clinical questions, as well as questions regarding va01rious aspects that may lead to physical, mental, and social limitations.
It will take approximately fifteen minutes to complete the questionnaire. This study is free of charge, and your participation is voluntary. By agreeing, you allow the results to be published in scientific journals and presented at health-related events. Confidentiality regarding your identity will be maintained and preserved.
Risks: The risks involved are emotional, which may include feelings, discomfort, tension, or fear related to the subject matter.
Risk Management: You have the right to withdraw from the study at any time by Yesply clicking "exit" or closing the questionnaire tab.
Risks inherent to the virtual environment: These involve limitations in ensuring complete confidentiality of data. To minimize these risks, your data will be stored on REDCap, an online data management platform with features designed to enhance the security of information storage.
Benefits: The benefits of this study include generating data to support the development of effective, integrated, sustainable, and evidence-based care strategies and public policies that prioritize health promotion and reduce the physical and mental impacts associated with long COVID. Additionally, it aims to contribute to strengthening efforts to combat this pandemic.
Confidentiality and Privacy
Autonomy: You have full autonomy in participating in this research. You may withdraw at any time and request the removal of your data without any penalty. Furthermore, you are guaranteed access to all information and clarifications about the study. You can request access at any time and contact the researchers through the contacts provided in this document.
Compensation and Indemnification: Your participation is voluntary, and there is no financial compensation. Your rights will be preserved and maintained. You are entitled to compensation from the researchers and the involved institutions in the event of any harm resulting from your participation, as per Resolutions 466/2012 and 510/2016.
Second Copy of the Online Informed Consent Form: You will have access to a second copy of this document. To do so, Yesply click on the link on the initial questionnaire page and download the document. It is important to keep a copy of this document for your records. Therefore, you must download and save the document. Any questions related to this procedure can be addressed by contacting the researchers.
If you have any questions, you may contact the Research Ethics Committee (CEP) of the Lauro Wanderley University Hospital of the Federal University of Paraíba, Rua Tabelião Stanislau Eloy, 585, Cidade Universitária, João Pessoa, or by phone at (83) 3206-0704 (Monday to Friday, business days, from 10 AM to 12 PM and from 2 PM to 4 PM) or with the responsible researchers (Renata Karina Reis - rkreis@eerp.usp.br; Ana Cristina de Oliveira e Silva - anacris.os@gmail.com; Glenda Roberta Oliveira Naiff Ferreira - grnaiff@gmail.com).
Your participation is completely voluntary, and if you wish to discontinue your participation, there will be no penalty. We also emphasize that you can request clarification about the study at any time. Your participation in the study is voluntary. To proceed, please choose one of the options below:

Do you agree to participate in this research?	◯ Yes	◯ No
**COVID-19 DIAGNOSIS**<br>Have you had a confirmed COVID-19 diagnosis through a laboratory test (rapid test or PCR)?	◯ Yes	◯ No

MODULE I: DEMOGRAPHIC CHARACTERIZATION	
In which state do you reside?	◯ Acre ◯ Alagoas ◯ Amapá ◯ Amazonas ◯ Bahia ◯ Ceara ◯ Distrito Federal ◯ Espirito Santo ◯ Goiás ◯ Maranhão ◯ Mato Grosso ◯ Mato Grosso do Sul ◯ Minas Gerais ◯ Pará ◯ Paraíba ◯ Paraná ◯ Pernambuco ◯Piauí ◯ Rio de Janeiro ◯ Rio Grande do Norte ◯ Rio Grande do Sul ◯ Rondônia ◯ Roraima ◯ Santa Catarina◯ São Paulo ◯Sergipe ◯Tocantins
City	
Where in the state do you live?	◯ Capital ◯ Urban area ◯ Rural area
Gender	◯Female ◯ Male
Do you belong to an indigenous group?	◯ Yes ◯ No
Do you belong to a riverside population?	◯ Yes ◯ No
Do you belong to a quilombola community?	◯ Yes ◯ No
Do you live with HIV?	◯ Yes ◯ No
What is your age (in full years)	
Height (cm)	
Weigth (kg)	
What is your highest level of education?	◯ No education/Never completed any grade ◯ Incomplete Elementary School ◯ Complete Elementary School ◯ Incomplete High School ◯ Complete High School ◯ Incomplete Higher Education (Undergraduate) ◯ Complete Higher Education (Undergraduate) ◯ Specialization ◯ Master's Degree ◯ Doctorate ◯ post-doctorate
What is your current occupation	Unemployed<br>Self-employed<br>Employed<br>Retired<br>Student
Are you a beneficiary of Government Social Programs? (e.g., Bolsa Família)	◯ Yes ◯ No
Individual income (in minimum wages, current amount: R$ 1,320.00)	◯ Less than 1 minimum wage ◯ 1 minimum wage ◯ 2 minimum wages ◯ 3 minimum wages ◯ 4 minimum wages ◯ 5 minimum wages ◯ 6 minimum wages ◯ 7 minimum wages ◯ 8 minimum wages ◯ 9 minimum wages ◯ 10 or more minimum wages
Skin Color	◯ White ◯ Black ◯ Brown ◯ Yellow ◯ Indigenous
Marital status	◯ Single ◯ Married ◯ Divorced/Separated ◯ In a stable union/Living together ◯ Widowed
**MODULE II: INFORMATION REGARDING SARS-COV-2 INFECTION**	
How many times have you had a confirmed diagnosis of COVID-19?	◯ 1 ◯ 2 ◯ 3 ◯ 4 ◯ 5 or more

In which year did you receive your first confirmed laboratory diagnosis of COVID-19?	◯ 2020 ◯ 2021 ◯ 2022 ◯ 2023	
In which year did you receive your second confirmed laboratory diagnosis of COVID-19?	◯ 2020 ◯ 2021 ◯ 2022 ◯ 2023	
In which year did you receive your third confirmed laboratory diagnosis of COVID-19?	◯ 2020 ◯ 2021 ◯ 2022 ◯ 2023	
In which year did you receive your fourth confirmed laboratory diagnosis of COVID-19?	◯ 2020 ◯ 2021 ◯ 2022 ◯ 2023	
In which year did you receive your fifth confirmed laboratory diagnosis of COVID-19?	◯ 2020 ◯ 2021 ◯ 2022 ◯ 2023	
Have you received any COVID-19 vaccines?	◯ Yes ◯ No	
How many doses?	◯ 1 ◯ 2 ◯ 3 ◯ 4 ◯ 5 or more	
Did you receive a COVID-19 diagnosis before being vaccinated?	◯ Yes ◯ No	
Before having COVID-19, were you diagnosed with any chronic disease?	◯ Yes ◯ No ◯ Not sure	
Which chronic diseases?	Yes	No
Cancer	◯	◯
Heart disease (heart attack, heart failure)	◯	◯
Hypertension	◯	◯
Obesity	◯	◯
Chronic kidney disease (kidney failure)	◯	◯
Chronic liver disease (cirrhosis, hepatitis)	◯	◯
Chronic lung disease (asthma, bronchitis, chronic obstructive pulmonary disease - COPD)	◯	◯
Stroke (Cerebrovascular Accident - CVA)	◯	◯
Diabetes	◯	◯
Transplants	◯	◯
Depression or anxiety	◯	◯
Tuberculosis	◯	◯
HIV	◯	◯
Were you using ART (Antiretroviral Therapy)?	◯ Yes ◯ No	
Thinking about your most severe COVID-19 infection, how would you classify it?	◯ Mild: I had respiratory symptoms but did not develop pneumonia or shortness of breath.<br>◯ Moderate: I had confirmed pneumonia, OR was hospitalized, OR required oxygen support.<br>◯ Severe/Critical: I was admitted to the ICU OR had to be intubated.	
During this episode, how were you treated?	◯ I treated myself without seeing a healthcare professional ◯ I was treated at home with support from healthcare professionals via phone or internet ◯ I received care at a health post (Primary care) ◯ I received care at the emergency room or a UPA (Emergency care unit) ◯ I received care at an outpatient clinic ◯ I was hospitalized in a general ward ◯ I was admitted to an Intensive Care Unit (ICU) ◯ I received care at a private hospital (through insurance)	
Did you receive oxygen during this episode due to	◯ Yes ◯ No	

COVID-19?	
Were you intubated during this episode due to COVID-19?	◯ Yes ◯ No
Did you receive antibiotics?	◯ Yes ◯ No
If yes mark	◯ Prescribed by a healthcare professional ◯ Not prescribed by a healthcare professional
Did you receive antivirals?	◯ Yes ◯ No
	◯ Prescribed by a healthcare professional ◯ Not prescribed by a healthcare professional
Did you receive ivermectin?	◯ Yes ◯ No
	◯ Prescribed by a healthcare professional ◯ Not prescribed by a healthcare professional
Did you receive chloroquine?	◯ Yes ◯ No
	◯ Prescribed by a healthcare professional ◯ Not prescribed by a healthcare professional
Did you take homemade remedies (herbal tea or mixtures)?	◯ Yes ◯ No
	◯ Prescribed by a healthcare professional ◯ Not prescribed by a healthcare professional
Have you heard of the term Long COVID?	◯ Yes ◯ No
	◯ Prescribed by a healthcare professional ◯ Not prescribed by a healthcare professional
If yes, where did you hear about it?	◯ Internet/social media ◯ NGOs ◯ Friends/colleagues ◯ Healthcare professional ◯ Television ◯ School or College ◯ Workplace

**MODULE III: SYMPTOMS RELATED TO LONG COVID**		
In any of the episodes, did you experience any physical or mental health symptoms that persisted for 4 weeks or more after COVID-19?	◯ Yes ◯ No	
Which of these symptoms you had put 4 weeks or more due the COVID-19?	Yes	No
Pain	◯	◯
Fatigue(Tiredness)	◯	◯
Dizziness	◯	◯
Sweating(Sweat in excess)	◯	◯
Chills(Tremors, goosebumps)	◯	◯
Loss of weight	◯	◯
Edema (Swelling)	◯	◯
Difficulty of Erection (Dysfunction Erectile)	◯	◯
Loss of Libido	◯	◯
Nausea	◯	◯
Diarrhea	◯	◯
Anxiety	◯	◯
Lack of attention	◯	◯
Sleep disturbances (Insomnia)	◯	◯
Mood changes	◯	◯

Indisposition	◯	◯
Depression	◯	◯
Stress	◯	◯
Suicidal thoughts	◯	◯
Suicide attempt	◯	◯
Kidney failure	◯	◯
Joint pain	◯	◯
Muscle pain	◯	◯
Increased heart rate	◯	◯
Palpitations (Pounding heartbeat)	◯	◯
Arrhythmia	◯	◯
Hypertension (High blood pressure)	◯	◯
Thrombosis	◯	◯
Pulmonary embolism	◯	◯
Heart attack	◯	◯
Stroke (Cerebrovascular Accident - CVA)	◯	◯
Loss of memory	◯	◯
Loss of smeel	◯	◯
Loss of taste	◯	◯
Hearing loss	◯	◯
Chest pain (when breathing)	◯	◯
Lack of air	◯	◯
Cough	◯	◯
Fibrosis pulmonary	◯	◯
Fall of hair Skin	◯	◯
Dermatitis	◯	◯
Changes menstrual	◯	◯
Other symptoms	◯ Yes ◯ No	
Which one(s)		
**MODULE IV: USE OF HEALTHCARE SERVICES**		
Did you seek any healthcare service to investigate and/or treat these symptoms?	◯ Yes ◯ No	
Which service did you seek?	◯ Public Health System (SUS) ◯ Private with insurance ◯ Private	
Where did you seek care?	◯ Health Post or Basic Health Unit ◯ Emergency Care Unit (UPA) ◯ Outpatient Clinic ◯ Hospitals ◯ University Projects	
Were you hospitalized for any of these symptoms?	◯ Yes ◯ No	
How long were you hospitalized?	◯ Less than a week ◯ One week ◯ Two weeks ◯ Three weeks ◯ One month or longer	
After recovering from COVID-19, did you continue to experience any symptoms?	◯ Yes ◯ No	
**MODULE V: LIFESTYLE HABITS**		
In your life, which of these substances have you used? (Note: only non-medically prescribed use).		
Tobacco/Cigarettes	◯ Yes ◯ No	
If yes, how often:	◯ Once or twice ◯ Monthly ◯ Weekly ◯ Daily or almost every day	
During the pandemic, did the use of this substance increase?	◯ Yes ◯ No	
Alcoholic beverages	◯ Yes ◯ No	
If yes, how often:	◯ Once or twice ◯ Monthly ◯ Weekly	

	◯ Daily or almost every day
During the pandemic, did the use of this substance increase?	◯ Yes ◯ No
Sedatives	◯ Yes ◯ No
If yes, how often:	◯ Once or twice ◯ Monthly ◯ Weekly<br>◯ Daily or almost every day
During the pandemic, did the use of this substance increase?	◯ Yes ◯ No
Marijuana	◯ Yes ◯ No
If yes, how often:	◯ Once or twice ◯ Monthly ◯ Weekly<br>◯ Daily or almost every day
During the pandemic, did the use of this substance increase?	◯ Yes ◯ No
Cocaine (Powder)	◯ Yes ◯ No
If yes, how often:	◯ Once or twice ◯ Monthly ◯ Weekly<br>◯ Daily or almost every day
During the pandemic, did the use of this substance increase?	◯ Yes ◯ No
Ecstasy (Pills)	◯ Yes ◯ No
If yes, how often:	◯ Once or twice ◯ Monthly ◯ Weekly<br>◯ Daily or almost every day
During the pandemic, did the use of this substance increase?	◯ Yes ◯ No
Crack	◯ Yes ◯ No
If yes, how often:	◯ Once or twice ◯ Monthly ◯ Weekly<br>◯ Daily or almost every day
During the pandemic, did the use of this substance increase?	◯ Yes ◯ No
Do you use any other substances not listed above?	◯ Yes ◯ No
Which ones?	
If yes, how often:	◯ Once or twice ◯ Monthly ◯ Weekly<br>◯ Daily or almost every day
During the pandemic, did the use of this substance increase?	◯ Yes ◯ No

MODULE VI: WHODAS 2.0
Think about the last 7 days and indicate from 0 to 4 according to the indication on the side, how much difficulty you had with the following:

	No difficulty	Mild difficulty	Moderate difficulty	Severe difficulty	Extreme difficulty or unable to do
Standing for long periods, such as 30 minutes	◯	◯	◯	◯	◯
Taking care of your household responsibilities	◯	◯	◯	◯	◯
Learning a new task, for example, learning how to get to a new place	◯	◯	◯	◯	◯
Participating in community activities (for example, celebrations, religious events, others)?	◯	◯	◯	◯	◯
Being emotionally affected by your health problems	◯	◯	◯	◯	◯

Focusing on doing something for ten minutes	○	○	○	○	○
Walking a long distance, such as one kilometer (or equivalent)?	○	○	○	○	○
Washing your whole body	○	○	○	○	○
Getting dressed	○	○	○	○	○
Dealing with people you do not know	○	○	○	○	○
Maintaining a friendship	○	○	○	○	○
Your daily work/school routine	○	○	○	○	○
How do you rate your ability to care for yourself after the pandemic compared to before COVID-19?	○ The same as before COVID-19 ○ Worse ○ Better				
TOTAL					

Compared to before COVID-19?	Better	Worse	The same as before COVID-19
Standing for long periods, such as 30 minutes	○	○	○
Taking care of your household responsibilities	○	○	○
Learning a new task, for example, learning how to get to a new place	○	○	○
Participating in community activities (for example, celebrations, religious events, others)?	○	○	○
Being emotionally affected by your health problems	○	○	○
Focusing on doing something for ten minutes	○	○	○
Walking a long distance, such as one kilometer (or equivalent)?	○	○	○
Washing your whole body	○	○	○
Getting dressed	○	○	○
Dealing with people you do not know	○	○	○
Maintaining a friendship	○	○	○
Your daily work/school routine	○	○	○
How do you rate your ability to care for yourself after the pandemic compared to before COVID-19?	○	○	○

**MODULE VII: FEMALE AND MALE SEXUAL QUOTIENT SCALE**	
You have an active sex life	○ Yes ○ No
Do you believe that after your COVID-19 diagnosis there were changes in your sexual practices?	○ Yes ○ No
How would you evaluate this change?	○ Greatly improved ○Improved somewhat ○

	No difference ◯ Worsened somewhat ◯ Worsened greatly
After your COVID-19 diagnosis, did you experience a change in the frequency of masturbation?	◯ Greatly improved ◯Improved somewhat ◯ No difference ◯ Worsened somewhat ◯ Worsened greatly
After your COVID-19 diagnosis, was there a change in the consumption of alcohol and/or other drugs before or during sexual activities?	◯ Greatly improved ◯Improved somewhat ◯ No difference ◯ Worsened somewhat ◯ Worsened greatly
After your COVID-19 diagnosis, was there a change in the consumption of pornography?	◯ Greatly improved ◯Improved somewhat ◯ No difference ◯ Worsened somewhat ◯ Worsened greatly
After your COVID-19 diagnosis, did you notice a change in the frequency of your sexual relations?	◯ Greatly improved ◯Improved somewhat ◯ No difference ◯ Worsened somewhat ◯ Worsened greatly
After your COVID-19 diagnosis, how would you assess your sexual performance? For example, was there a reduction in time and/or did you feel more fatigued during sexual relations?	◯ Greatly improved ◯Improved somewhat ◯ No difference ◯ Worsened somewhat ◯ Worsened greatly
**Please answer this questionnaire honestly, based on the last six months (FEM):**	
Do you often think spontaneously about sex, remember sex, or imagine yourself having sex?	◯ Never ◯ Rarely ◯ Sometimes ◯ About 50% of the time ◯ Most of the time ◯ Always
Is your interest in sex sufficient for you to engage in sexual relations willingly?	◯ Never ◯ Rarely ◯ Sometimes ◯ About 50% of the time ◯ Most of the time ◯ Always
Do foreplay activities (caresses, kisses, hugs, etc.) stimulate you to continue the sexual relationship?	◯ Never ◯ Rarely ◯ Sometimes ◯ About 50% of the time ◯ Most of the time ◯ Always
Do you usually get lubricated (wet) during sexual relations?	◯ Never ◯ Rarely ◯ Sometimes ◯ About 50% of the time ◯ Most of the time ◯ Always
During sexual relations, as your partner's excitement increases, do you also feel more stimulated for sex?	◯ Never ◯ Rarely ◯ Sometimes ◯ About 50% of the time ◯ Most of the time ◯ Always
During sexual relations, do you relax your vagina enough to facilitate penetration?	◯ Never ◯ Rarely ◯ Sometimes ◯ About 50% of the time ◯ Most of the time ◯ Always
Do you usually feel pain during sexual relations when the penis penetrates your vagina?	◯ Never ◯ Rarely ◯ Sometimes ◯ About 50% of the time ◯ Most of the time ◯ Always
Can you stay engaged without getting distracted (without losing concentration) during sexual relations?	◯ Never ◯ Rarely ◯ Sometimes ◯ About 50% of the time ◯ Most of the time ◯ Always
Are you able to reach orgasm (maximum pleasure) during the sexual relations you engage in?	◯ Never ◯ Rarely ◯ Sometimes ◯ About 50% of the time ◯ Most of the time ◯ Always
Does the satisfaction you gain from sexual relations make you want to have sex again on other days?	◯ Never ◯ Rarely ◯ Sometimes ◯ About 50% of the time ◯ Most of the time ◯ Always
**Please answer this questionnaire honestly, based on the last six months (MALE):**	
Is your interest in sex sufficient for you to want to initiate sexual activity?	◯ Never ◯ Rarely ◯ Sometimes ◯ About 50% of the time ◯ Most of the time ◯ Always
Does your seductive ability give you the confidence to engage in sexual pursuit?	◯ Never ◯ Rarely ◯ Sometimes ◯ About 50% of the time ◯ Most of the time ◯ Always
Are the preliminaries of your sexual activity enjoyable and satisfying for you and your partner?	◯ Never ◯ Rarely ◯ Sometimes ◯ About 50% of the time ◯ Most of the time ◯ Always
Does your sexual performance vary depending	◯ Never ◯ Rarely ◯ Sometimes ◯ About 50%

on whether your partner is able to be satisfied during the sexual act with you?	of the time ◯ Most of the time ◯ Always
Can you maintain an erection long enough to complete sexual activity with satisfaction?	◯ Never ◯ Rarely ◯ Sometimes ◯ About 50% of the time ◯ Most of the time ◯ Always
After sexual stimulation, is your erection sufficiently firm to ensure a satisfactory sexual relation?	◯ Never ◯ Rarely ◯ Sometimes ◯ About 50% of the time ◯ Most of the time ◯ Always
Are you able to achieve and maintain the same quality of erection in various sexual relations you engage in on different days?	◯ Never ◯ Rarely ◯ Sometimes ◯ About 50% of the time ◯ Most of the time ◯ Always
Can you control ejaculation so that your sexual activity lasts as long as you wish?	◯ Never ◯ Rarely ◯ Sometimes ◯ About 50% of the time ◯ Most of the time ◯ Always
Are you able to reach orgasm during the sexual relations you engage in?	◯ Never ◯ Rarely ◯ Sometimes ◯ About 50% of the time ◯ Most of the time ◯ Always
Does your sexual performance encourage you to have sex again in other opportunities?	◯ Never ◯ Rarely ◯ Sometimes ◯ About 50% of the time ◯ Most of the time ◯ Always
**MODULE VIII: QUALITY OF LIFE**<br>Please click the box that best describes your health **TODAY.**	
MOBILITY	◯ I have no problems walking<br>◯ I have slight problems walking<br>◯ I have moderate problems walking<br>◯ I have severe problems walking<br>◯ I am unable to walk
SELF-CARE	◯ I have no problems washing or dressing myself ◯ I have slight problems washing or dressing myself<br>◯ I have moderate problems washing or dressing myself<br>◯ I have severe problems washing or dressing myself<br>◯ I am unable to wash or dress myself
USUAL ACTIVITIES (e.g. work, study, household chores, family or leisure activities)	◯ I have no problems doing my usual activities<br>◯ I have slight problems doing my usual activities<br>◯ I have moderate problems doing my usual activities<br>◯ I have severe problems doing my usual activities<br>◯ I am unable to do my usual activities
PAIN / DISCOMFORT	◯ I have no pain or discomfort<br>◯ I have slight pain or discomfort<br>◯ I have moderate pain or discomfort<br>◯ I have severe pain or discomfort<br>◯ I have extreme pain or discomfort
ANXIETY / DEPRESSION	◯ I am not anxious or depressed<br>◯ I am slightly anxious or depressed<br>◯ I am moderately anxious or depressed<br>◯ I am very anxious or depressed<br>◯ I am extremely anxious or depressed
We would like to know how good or bad your health is **TODAY.**<br>This scale is numbered from 0 to 100. 100 means the best health you can imagine. 0 means the worst health you can imagine. Please click on the scale to indicate how your health is TODAY.<br>0 ____ 100	

MODULE IX: DASS-21

Please carefully read each of the statements below and indicate how much it applied to you during the past week, according to the following scale:

	Did not apply at all	Applied to some extent, or for a short time	Applied to a considerable degree, or for a good part of the time	Applied a lot, or most of the time
I had difficulty calming myself down	○	○	○	○
I was aware that my mouth was dry	○	○	○	○
I seemed unable to experience any positive feelings	○	○	○	○
I had difficulty breathing (e.g., excessively fast breathing, shortness of breath, in the absence of physical exertion)	○	○	○	○
I had difficulty taking initiative to do things	○	○	○	○
I tended to overreact to situations	○	○	○	○
I experienced trembling (e.g., in my hands)	○	○	○	○
I felt generally very nervous	○	○	○	○
I was worried about situations where I might panic and appear ridiculous	○	○	○	○
I felt like I had nothing to look forward to	○	○	○	○
I felt agitated	○	○	○	○
I had difficulty relaxing	○	○	○	○
I felt downhearted	○	○	○	○
I was intolerant of things that prevented me from continuing what I was doing	○	○	○	○
I felt like I was going to panic	○	○	○	○
I couldn’t get excited about anything	○	○	○	○
I felt like I wasn’t worth much as a person	○	○	○	○
I felt sensitive	○	○	○	○

I was aware of the functioning/heartbeat of my heart in the absence of physical effort (e.g., feeling of increased heart rate, cardiac arrhythmia)	○	○	○	○
I felt scared for no good reason	○	○	○	○

To answer the following questions, consider the past three months:

	Never	Rarely	Occasionally	Frequently	Always
Have you had a strong and constant feeling of lacking energy?	○	○	○	○	○
Have you noticed that you need more energy to handle your daily tasks?	○	○	○	○	○
Have you felt unwilling to do things?	○	○	○	○	○
Have you woken up feeling exhausted and worn out?	○	○	○	○	○
Have you needed to rest more?	○	○	○	○	○
Have you been able to do your daily activities?	○	○	○	○	○
Has your interest in sex or your desire to engage in sexual activity decreased?	○	○	○	○	○
Has it been more difficult to focus on something for a long time?	○	○	○	○	○

To answer the following four items, consider the past three months:

	Almost never	Occasionally	Often	Very frequently	Almost always
I look for creative ways to overcome difficult situations	○	○	○	○	○
Regardless of what happens to me, I believe I can control my reactions	○	○	○	○	○
I believe I can grow positively by dealing with difficult situations	○	○	○	○	○
I actively seek ways to replace the losses I encounter in life	○	○	○	○	○
